# Transbronchial Cryoablation for Early‐Stage Non‐Small Cell Lung Cancer of the Central Airway Complicated by Idiopathic Pulmonary Fibrosis

**DOI:** 10.1002/rcr2.70408

**Published:** 2025-11-18

**Authors:** Masamitsu Hamakawa, Takashi Niwa, Ryoju Sato, Yasushi Fukuda, Toshihide Yokoyama, Tadashi Ishida

**Affiliations:** ^1^ Department of Respiratory Medicine Kurashiki Central Hospital Okayama Japan; ^2^ Department of Respiratory Medicine Kanagawa Cardiovascular and Respiratory Center Kanagawa Japan

**Keywords:** bronchoscopy, cryoablation, interstitial lung disease, lung cancer, transbronchial cryoablation

## Abstract

Interstitial lung disease presents significant challenges in managing early‐stage non‐small cell lung cancer due to the high risk of acute exacerbation with standard therapies. We report the case of an 82‐year‐old man with idiopathic pulmonary fibrosis and central airway squamous cell carcinoma who was treated with transbronchial cryoablation. The procedure was completed without major complications, and no tumour recurrence was observed over an 11‐month follow‐up. This case highlights the potential of transbronchial cryoablation as a feasible local treatment option for early‐stage non‐small cell lung cancer in patients with idiopathic pulmonary fibrosis, offering a safer alternative to surgery or radiation therapy.

## Graphical abstract

We report the case of an 82‐year‐old man with idiopathic pulmonary fibrosis and central airway squamous cell carcinoma successfully treated with transbronchial cryoablation. The procedure was safe, with no recurrence over 11 months, suggesting its potential as a local treatment alternative to surgery or radiation in patients with lung cancer and interstitial lung disease.

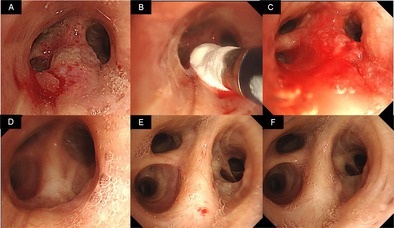

## 1 Introduction

Interstitial lung disease (ILD) remains challenging due to its poorly understood mechanisms and the lack of well‐established treatment strategies [1]. Therapeutic decision‐making is particularly difficult in patients with ILD who develop early‐stage non‐small cell lung cancer (NSCLC) [2]. Standard treatment modalities, including surgical resection, radiation therapy and chemotherapy, carry a substantial risk of triggering acute exacerbation of the underlying ILD [2].

Against this background, bronchoscopic approaches have been explored as alternative local treatment options for high‐risk patients [3, 4, 5, 6]. Among these, transbronchial cryoablation has recently attracted attention as a useful technique [4, 5]. Although not a new modality, this approach can be performed relatively easily without the need for specialised equipment and is reportedly effective in achieving local control of centrally located airway lesions. Importantly, the ability to perform the treatment in patients with ILD who are unsuitable for surgery or stereotactic radiotherapy is clinically significant.

We have previously reported successful application of this method in patients with recurrent lung cancer following surgery [4]. Building upon our experience, we applied this technique to a patient with early‐stage NSCLC complicated by idiopathic pulmonary fibrosis (IPF).

## 2 Case Report

An 82‐year‐old man was referred to our hospital from a local clinic. He presented with ILD and chronic respiratory failure and required supplemental oxygen at a flow rate of 3 L/min. His past medical history included gastroesophageal reflux disease, hypertension and diabetes mellitus. His performance status was grade 2 according to the Eastern Cooperative Oncology Group scale. Pulmonary function tests showed a percentage predicted forced vital capacity of 64.3% and a percentage predicted diffusing capacity for carbon monoxide of 46.6%. Arterial blood gas analysis under supplemental oxygen at 3 L/min revealed: PaO_2_, 72.4 mmHg; PaCO_2_, 40.9 mmHg and pH of 7.38. These results were consistent with chronic respiratory failure. Results of the initial laboratory tests, including haematological, biochemical and serological data, are summarised in Table 1.

**TABLE 1:** Laboratory data.

Parameter	Result	Reference range
Haematology		
WBC	6200/μL	3300–8600/μL
Neut	66.5%	40%–70%
Lymp	19.6%	20%–45%
Eos	3.9%	0%–6%
Baso	1.1%	0%–1%
Mono	8.4%	2%–10%
Hb	12.2 g/dL	13.5–17.5 g/dL
RBC	392 × 10^4^/μL	435–555 × 10^4^/μL
Biochemistry		
CRP	0.53 mg/dL	0–0.14 mg/dL
TP	7.6 g/dL	6.6–8.1 g/dL
Alb	3.3 g/dL	4.1–5.1 g/dL
AST	57 U/L	13–30 U/L
ALT	30 U/L	10–42 U/L
LDH	302 U/L	124–222 U/L
BUN	20 mg/dL	8–20 mg/dL
Cr	1.15 mg/dL	0.65–1.07 mg/dL
Serology		
MPO‐ANCA	< 1.0 U/mL	0.0–3.4 U/mL
Anti‐CCP	< 0.5 U/mL	< 4.5 U/mL
ANA	Positive (1:40)	< 1:40
Avian‐specific IgG antibody	Negative	Negative
KL‐6	1430 U/mL	105–401 U/mL
SP‐D	228.2 ng/mL	0.0–109.8 ng/mL
CYFRA	4.3 ng/mL	< 3.5 U/mL

**FIGURE 1:**
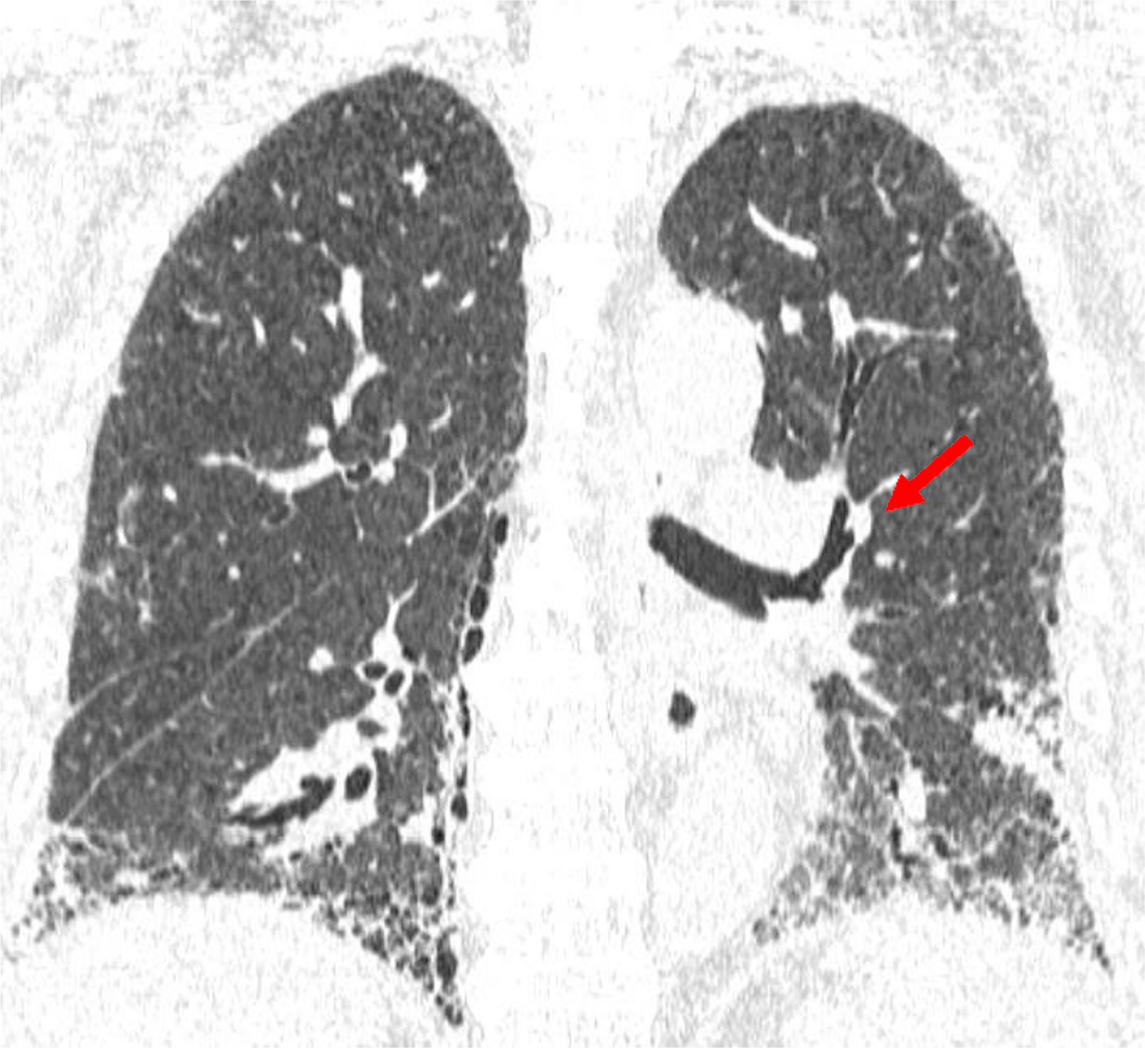
Computed tomography findings before transbronchial cryoablation. Computed tomography shows interstitial pneumonia with a subpleural and basal predominance, as well as a nodule located in the superior segmental bronchus of the left lung (arrow).

**FIGURE 2:**
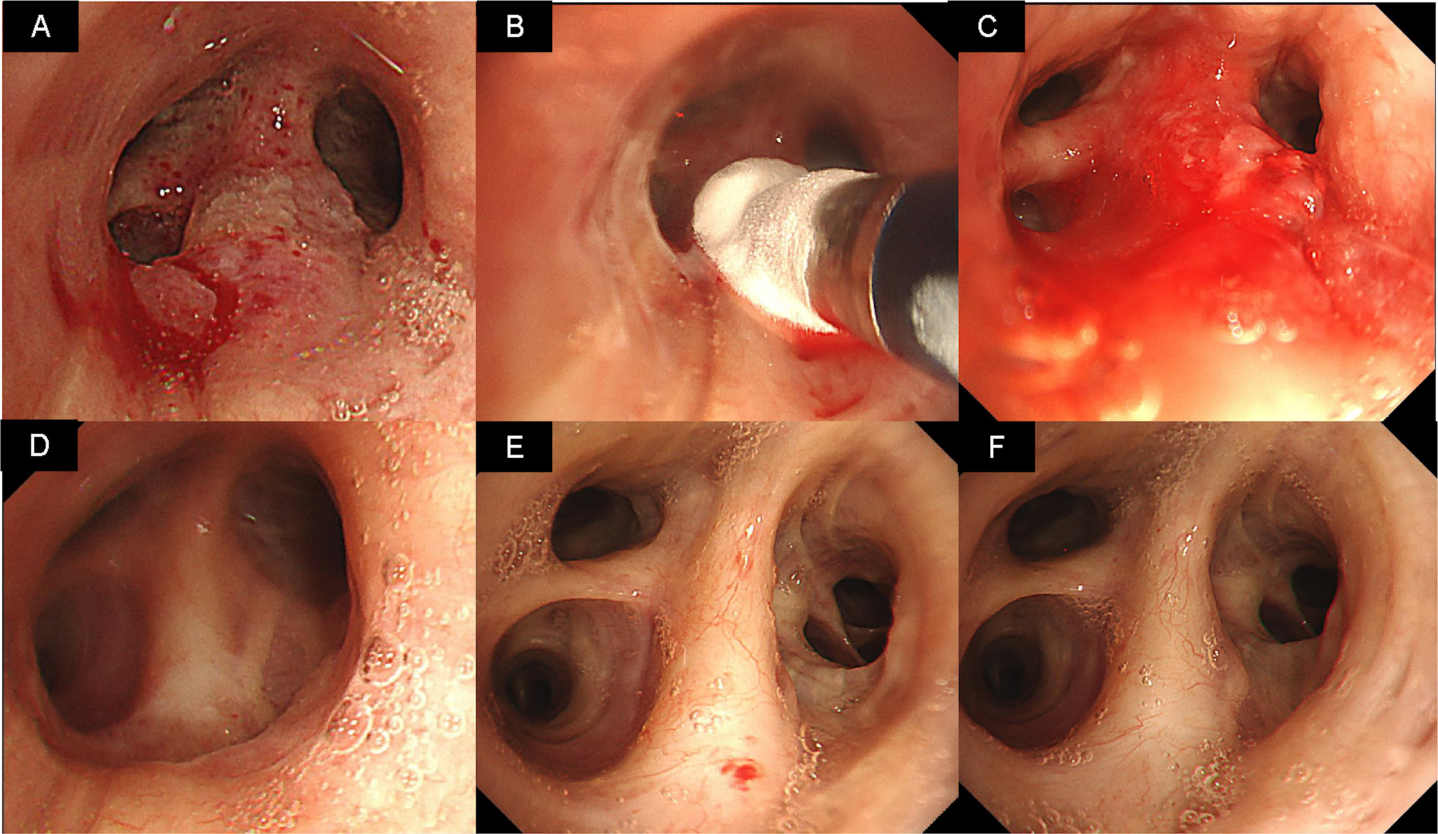
Changes in bronchoscopy findings during the treatment of squamous cell carcinoma in the superior segmental bronchus of the left lung. (A) Bronchoscopy shows a hyperemic, friable tumour in the superior segmental bronchus of the left lung. Histopathological examination of tumour tissue obtained by biopsy identified squamous cell carcinoma. (B) Bronchoscopy findings during transbronchial cryoablation. The flexible cryoprobe was applied three times for 30 s each cycle for a visible lesion. (C) Bronchoscopy findings immediately after transbronchial cryoablation. Bronchoscopy shows mild bleeding and oedema at the treatment site. (D) Bronchoscopy findings after transbronchial cryoablation. One month after transbronchial cryoablation, bronchoscopy shows post‐treatment scarring, with no clear evidence of tumour recurrence. (E) Bronchoscopy findings after transbronchial cryoablation. Three months after transbronchial cryoablation, progressive regeneration of the bronchial epithelium is observed, with no evident tumour recurrence. (F) Bronchoscopy findings after transbronchial cryoablation. Eleven months after transbronchial cryoablation, bronchoscopy shows no evident tumour recurrence.

Computed tomography revealed subpleural‐ and basal‐predominant fibrotic changes, consistent with ILD, which was subsequently diagnosed as IPF. In addition, a nodule was identified in the superior segmental bronchus of the left lung (Figure 1). Bronchoscopy revealed a hyperemic, friable tumour in the superior segmental bronchus of the left lung (Figure 2A). Histopathological examination of biopsy specimens confirmed the diagnosis of squamous cell carcinoma. Positron emission tomography‐computed tomography and brain magnetic resonance imaging showed no evidence of metastasis. Given the location of the tumour in the central airway, there was a potential risk of haemoptysis if left untreated. However, due to comorbidities and the poor general condition of the patient, neither surgery nor radiotherapy was performed. Although photodynamic therapy (PDT) is an approved treatment option in Japan for centrally located airway tumours, the location of the nearest facility was far from where the patient resided and the patient preferred to avoid frequent travel due to the physical burden. Transbronchial cryoablation was therefore performed as a minimally invasive local therapy to reduce the risk of haemoptysis, using a flexible cryoprobe applied to all visible lesions three times for 30‐s cycles, in accordance with a previously reported technique (Figure 2B) [4].

Bronchoscopy immediately after the procedure detected mild bleeding and oedema at the treatment site, without major complications (Figure 2C). Follow‐up bronchoscopy performed 1 month later showed post‐treatment scarring with no clear evidence of tumour recurrence (Figure 2D). At 3 months post‐treatment, progressive regeneration of the bronchial epithelium was observed and no recurrent lesion was identified (Figure 2E). Accordingly, treatment with nintedanib at a dose of 300 mg/day was initiated. Follow‐up bronchoscopy at 11 months after cryoablation revealed no evident tumour recurrence at the previous treatment site (Figure 2F). Cytological examination by brush sampling was performed at each follow‐up bronchoscopy, and all results were negative for malignancy. Ongoing outpatient surveillance is planned to monitor the clinical course of both IPF and lung cancer.

## 3 Discussion

Surgery is still the gold standard for early‐stage NSCLC, but outcomes remain far from ideal. Around 20%–30% of stage I patients experience recurrence, usually within 12–17 months after lobectomy [6, 7]. In addition, perioperative risk increases with age, poor performance status and reduced pulmonary function [6]. These risks become particularly severe in patients with concomitant ILD, notably IPF, where surgery may provoke postoperative acute exacerbations with considerable mortality, particularly among those with honeycombing [2]. For medically inoperable patients, stereotactic ablative radiotherapy (SABR) serves as the standard definitive local therapy [6, 8]. Although randomised trials comparing SABR with surgery are limited, the available data consistently demonstrate a more favourable safety profile for SABR [6]. Moreover, meta‐analyses of non‐randomised studies have suggested comparable outcomes after adjusting for competing risks related to age and comorbidities, while other reports have indicated superior long‐term survival with lobectomy [6]. Nevertheless, concerns regarding severe toxicity have historically limited the use of SABR in patients with ILD [2, 8]. Palma et al. [8] prospectively evaluated 39 patients with early‐stage NSCLC complicated by ILD, reporting survival and toxicity outcomes that support SABR as a considered option when risks and benefits are carefully weighed. Even so, the potential for acute exacerbation and the lack of truly safe local options in this population underscore the need for alternative, less‐invasive approaches. In this context, bronchoscopic modalities have attracted attention as emerging local therapies that can achieve tumour control without surgery or radiation [4, 5, 6]. However, bronchoscopic modalities also have inherent limitations. PDT has been used selectively in Japan, but carries several drawbacks, including a risk of phototoxic reactions and the need for specialised equipment, light sources and strict drug‐handling protocols, which restrict the availability of this method to a limited number of hospitals [3, 6]. Further, heat‐based ablation modalities such as bronchoscopic laser interstitial thermal therapy and bronchoscopic microwave ablation are generally avoided under oxygen‐enriched conditions, as delivery of thermal energy in an oxygen‐rich airway increases the risk of airway fire and thermal injury [6]. By contrast, transbronchial cryoablation achieves non‐combustive tissue destruction without increasing intraluminal oxygen demand and can be performed using purely bronchoscopic workflows [4, 5]. Therefore, this method can be safely and effectively applied even in patients requiring oxygen therapy for chronic respiratory failure associated with ILD, representing a promising alternative to conventional endoscopic treatments.

This case report showed several limitations that warrant consideration. First, transbronchial cryoablation may be most suitable for endobronchial tumours that can be directly visualised and contacted with the cryoprobe. In contrast, extrinsic tumours may not be adequately treated using this approach, restricting its generalisability. Second, several procedural parameters remain undefined, including the optimal freezing time, number of cycles and the safety of overlapping freeze zones. In addition, the influence of tumour location within the airway tree, the clinical history such as prior thoracic treatments, and the anatomical relationship of the tumour to critical airway structures were likely to have affected both safety and efficacy, yet these factors have not been systematically studied. Third, the present case was followed for a relatively short duration of 11 months post‐procedure, and long‐term outcomes over a multi‐year timeframe remain unknown. Finally, in patients with underlying interstitial lung disease (ILD) who are receiving corticosteroid therapy, the risk of complications at the cryoablation site may conceivably be increased, although this has yet to be formally assessed.

In conclusion, transbronchial cryoablation deserves consideration as a local treatment for early‐stage lung cancer of the central airway complicated by interstitial pneumonia.

## Author Contributions

M.H. wrote the manuscript. T.N., R.S., Y.F., T.Y. and T.I. contributed to data collection. All authors read and approved the final manuscript.

## Consent

The authors declare that written informed consent was obtained for the publication of this manuscript and accompanying images using the form provided by the Journal.

## Conflicts of Interest

The authors declare no conflicts of interest.

## Data Availability

Research data are not shared.
